# Butane Tetracarboxylic Acid Grafted on Polymeric Nanofibrous Aerogels for Highly Efficient Protein Absorption and Separation

**DOI:** 10.3390/polym16091270

**Published:** 2024-05-02

**Authors:** Jianwei Lu, Yangang Jiang, Yufei Qiao, Zihao Wen, Zhengjin Luo, Mukhtar Ahmed, Amjad Ali, Li Guo

**Affiliations:** 1 School of Materials Science and Engineering, Jiangsu University, Zhenjiang 212013, China; 2212105014@stmail.ujs.edu.cn (Y.J.); yfqiao0104@163.com (Y.Q.); m19834996999@163.com (Z.W.); 18406801063@163.com (Z.L.); amjadali@zju.edu.cn (A.A.); 2 Department of Zoology, College of Science, King Saud University, P.O. Box 2455, Riyadh 11451, Saudi Arabia; mahmed1@ksu.edu.sa

**Keywords:** protein purification, absorption and separation, impregnation processes, nanofibrous aerogels, abundant absorption ligands

## Abstract

Developing high-performance and low-cost protein purification materials is of great importance to meet the demands for highly purified proteins in biotechnological industries. Herein, a facile strategy was developed to design and construct high-efficiency protein absorption and separation media by combining aerogels’ molding techniques and impregnation processes. Poly (ethylene-co-vinyl alcohol) (EVOH) nanofibrous aerogels (NFAs) were modified by grafting butane tetracarboxylic acid (BTCA) over them in situ. This modification was carried out using polyphosphoric acid as a catalyst. The resulting EVOH/BTCA NFAs exhibited favorable comprehensive properties. Benefiting from the highly interconnected porous structure, good underwater compressive properties, and abundant absorption ligands, the obtained EVOH/BTCA NFAs possessed a high static absorption capacity of 1082.13 mg/g to lysozyme and a short absorption equilibrium time of about 6 h. A high saturated dynamic absorption capacity for lysozyme (716.85 mg/g) was also realized solely by gravity. Furthermore, EVOH/BTCA NFAs displayed excellent reusability, good acid and alkaline resistance, and unique absorption selectivity performance. The successful synthesis of such aerogels can provide a potential candidate for next-generation protein absorbents for bio-separation and purification engineering.

## 1. Introduction

The purified protein is crucial for advancing life science technologies and has found extensive use in immunotherapy, medicinal therapy, food industry, and health detection [1,2,3]. Protein absorption and separation has been demonstrated to be one of the most efficient methods for obtaining highly purified proteins due to its high precision and convenient operation [4,5,6]. Currently, the conventional absorption and separation process is proceeded by microparticle media-packed chromatography columns. However, those chromatography columns are generally limited by a relatively long retention time and low processing rate, which are caused by the tiny pores inside of the microparticle media [7,8]. In addition, the gradual accumulation of microparticle media under the rapid flow of liquid would cause significant pressure drop and energy consumption, restricting the further development of microparticle media in large-scale protein purification [9,10]. Alternatively, fibers have been developed that show potential in the construction of high-performance absorption media with a fast processing rate and low flow resistance [11]. Although several fibrous-based media have been successfully fabricated for protein purification, those materials normally exhibited poor absorption capacity caused by their big fiber diameters and insufficient absorption sites [12].

As a newly important material, nanofibers with features of super small diameter, high aspect ratio and big specific surface area serve as promising building blocks to fabricate absorption media [13,14]. To date, a series of nanofibers has been assembled into membranes for protein absorption. Amaly [15] chelated copper ions with carboxylated nylon 6 nanofibrous membranes (NFMs) to generate protein absorbents, and the obtained absorbents exhibited an adsorption capability of 220.00 mg/g with good selectivity and reusability. Chang [16] functionalized polyacrylonitrile NFMs with ethylene diamine and bromoacetic acid to form polyacid IEX NFMs with a lysozyme binding capacity of 305.33 mg/g. Zhou [17] modified silk fibroin/cellulose blend NFMs with sodium-3-sulfobenzoate under mild conditions for the adsorption of lysozyme (636.00 mg/g). However, some intrinsic limitations of two-dimensional (2D) membranes, such as small pore size, narrow pore channels, and difficulty in assembling large-sized chromatography columns, can cause unsatisfactory protein capture performance [18,19]. Alternatively, nanofibers also can be presented to construct aerogels with three-dimensional (3D) porous skeletal structures [20,21,22]. Nanofibrous aerogels (NFAs) possess high porosity, an interconnected pore structure, a tunable shape, and good size, which means that they have great promise for fabricating high-efficiency absorption media [23]. However, several bottlenecks still existed in aerogel media, that is, poor nanofiber preparation efficiency and a complicated media fabrication process. Therefore, the design and creation of NFA-based absorption media with favorable integrated properties is highly desired.

In this study, EVOH nanofibers obtained using the significant process exhibited melt-extrusion phase separation were taken to prepare 3D highly interconnected porous aerogels, then BTCA was introduced onto aerogels to form EVOH/BTCA NFAs under the catalysis of polyphosphoric acid. The morphologies, surface wettability, zeta potential, underwater compressive properties, and protein absorption performance were controlled by BTCA contents. Attributed to the abundant absorption ligands, EVOH/BTCA NFAs exhibited outstanding static (1082.13 mg/g) and dynamic (716.85 mg/g) protein absorption capacity. The effects of pH values, ionic strength and species, and protein species on the absorption properties of EVOH/BTCA NFAs were also investigated. We expect that the successful preparation of EVOH/BTCA NFAs could provide a promising strategy for developing protein absorption media with favorable comprehensive properties.

## 2. Materials and Methods

### 2.1. Materials

The Model 381-20 of cellulose acetate butyrate ester (CAB) was purchased from Eastman Chemical Company(Kingsport, TN, USA). We bought the EVOH Model ET3803 masterbatch from Nippon Synthetic Chemical Industry Co., Ltd.(Osaka, Japan). Glutaraldehyde aqueous solution (GA, 25 wt%), tert-butanol (>98%), acetic acid, polyphosphoric acid (PPA, phosphorus pentoxide content >85 wt%), butane tetracarboxylic acid (BTCA), acetone, phosphate buffer saline (PBS), phosphoric acid (H_3_PO_4_), NaOH, LiCl, KCl, MgCl_2_, NaCl, and NaSO_4_ were purchased from Sinopharm Chemical Reagent Co., Ltd. (Shanghai, China). Papain, lysozyme, ovalbumin, bromelain, bovine serum albumin, and pepsin were bought from Sangon Biotech Co., Ltd. (Shanghai, China).

### 2.2. Preparation of EVOH Nanofibrous Aerogels (NFAs)

EVOH nanofibers were prepared using the melt-extrusion phase separation method, and these nanofibers were subsequently utilized as the fundamental components for the fabrication of aerogels [24,25,26]. In brief, a 1 gm sample of EVOH nanofiber was evenly dispersed in a 100 mL dispersion of tert-butanol and water. This was achieved by homogenizing the mixture at 13,000 rpm, resulting in uniform EVOH nanofibrous dispersions. Glutaraldehyde aqueous solutions with different mass fractions were added into obtained nanofibrous dispersions with magnetic stirring. Dispersions were mixed with acetic acid solution to adjust the pH to 3–4. The composite nanofibrous dispersions were then freeze-dried to form frameworks. Ultimately, 75 °C was applied to the frameworks to prepare EVOH NFAs.

### 2.3. Fabrication of EVOH/BTCA NFAs

The impregnation method was applied to fabricated EVOH/BTCA NFAs. BTCA was selected as a modifying agent and dissolved into water to generate modified solutions (mass fraction: 0, 2, 4, 6, 8, 10, 12, 14 and 16 wt%). PPA was added into modified solutions as a catalyst. EVOH NFAs were soaked into modified solutions for 30 min, then removed and placed in a lyophilizer to be freeze-dried. EVOH/BTCA NFAs were obtained through the grafting polymerization process at 100 °C.

### 2.4. Instruments and Characterization

Utilizing a scanning electron microscope (Hitachi S-4800, SEM), the structure was studied. Attenuated total reflection–Fourier transform infrared (Nicolet 6700, ATR-FTIR) was employed to determine the chemical composition of the surface. The thermogravimetric analyzer (TA Q5000IR, TGA) was used for evaluating the thermal characteristics. A Nano Zetasizer (ZS 90) was used to characterize the zeta potential. The surface wettability was investigated by employing a contact angle meter (AM4111T). The mechanical properties were measured by employing a universal testing machine (Instron 3365). The cylindrical EVOH/BTCA NFAs had a diameter of around 20 mm and a height of approximately 20 mm. Using an ultraviolet-visible (UV-vis) spectrophotometer (UV-1700), the concentration of protein solutions was determined.

### 2.5. Testing Protein Absorption Performance

PBS was added into water to generate the PBS solutions with stirring. The protein solutions with various properties were formed through adding model proteins into PBS solutions. Then, 10 mg EVOH/BTCA NFAs were immersed in protein solutions to absorb protein for a period of time, then the EVOH/BTCA NFAs were moved out and cleaned to avoid non-specific absorption. The change in protein solutions was detected by employing a UV-vis spectrophotometer. The following formula calculated the absorption capacity of the EVOH/BTCA NFAs:

(1) $$Q=\frac{\left(C_{0}-C\right)V}{m}$$

where Q is the absorption capacity (mg/g), $C_{0}$ and C is the concentration of protein solution before and after absorption (mg/mL), respectively, V is the volume of protein solutions (mL), m is the weight of EVOH/BTCA NFAs (mg).

## 3. Results and Discussion

### 3.1. EVOH/BTCA NFA Preparation and Design

In order to design and prepare protein absorption materials with excellent performance, the aerogels were optimized on the basis of three requirements: (1) aerogels that are surface wettable should have high hydrophilicity to prevent non-specific binding; (2) the mechanical structure of EVOH/BTCA NFAs should be stable for long-term applications; (3) a large number of absorption ligands should be displayed on aerogels to achieve effective absorption. By employing EVOH nanofibers as building blocks, the first criterion was fulfilled. In order to satisfy the second requirement, glutaraldehyde was taken to bond nanofibers to improve the mechanical properties. As shown in Figure S1, the bonding among EVOH nanofibers was obviously observed, which was generated via polymerization between glutaraldehyde and EVOH nanofibers. The last requirement was satisfied by selecting BTCA as a modifier, giving aerogels with abundant absorption ligands [27,28].

**Figure 1:**
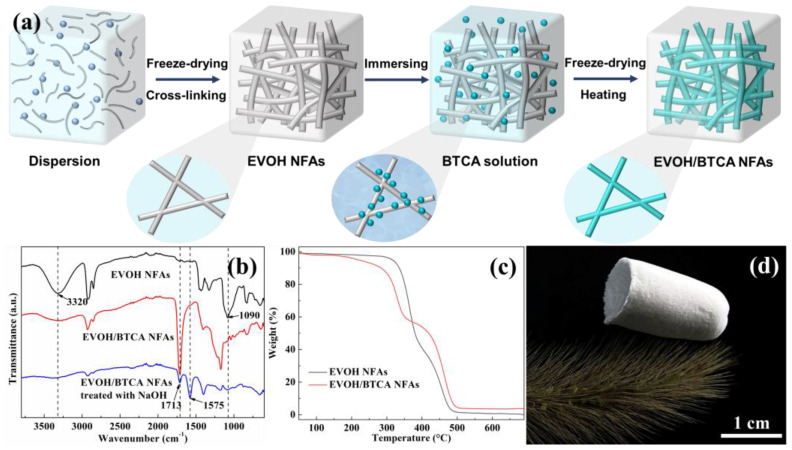
This figure shows the following: (**a**) a schematic explanation of the EVOH/BTCA NFA preparation method; (**b**) EVOH NFA ATR-FTIR spectra; (**c**) TGA curves of EVOH NFAs and EVOH/BTCA NFAs; (**d**) EVOH/BTCA NFAs standing on top of green bristlegrass.

The creation of EVOH NFAs, grafting of carboxyl groups, and dispersion of EVOH nanofibers are the three primary steps in the fabrication of EVOH/BTCA NFAs, as shown in Figure 1a. First, EVOH nanofibers were homogenized into a mixture of substances to create a nanofibrous dispersion. After that, EVOH nanofibers were chemically cross-linked and freeze-dried to create EVOH NFAs, which were then attached [25]. To achieve carboxylic grafting, the resulting EVOH NFAs were soaked into the modified solutions, dried, and heated. Water washing was used to remove the unreacted BTCA and PPA, and EVOH/BTCA NFAs were obtained through drying. The chemical compositions were confirmed via analysis of the ATR-FTIR spectra. As presented in Figure 1b, the peak at 1090 cm^−1^ was attributed to ether bonds generated from the reaction of EVOH with glutaraldehyde, indicating the successful cross-linking [29]. After modification, an absorption peak at 1713 cm^−1^ was attributed to C=O and the peak intensity at 3320 cm^−1^ (corresponding to -OH) decreased sharply, suggesting a reaction between EVOH nanofibers and BTCA. Both ester and carboxyl groups contain C=O, and NaOH solution treatment was used to identify them [30]. The new peak located at about 1575 cm^−1^ is the stretching vibration of carboxyl, demonstrating the carboxyl grafting on EVOH NFAs. The nanofiber surface grafting is shown in Figure S2. BTCA with abundant carboxyl groups were grafted onto nanofibers via esterification [31].

The thermal degradation of EVOH/BTCA NFA components was studied through TGA. The TGA curves of EVOH NFAs (Figure 1c) revealed two distinct and easily identifiable degradation phases. The initial degradation phase, occurring between about 296 °C and 385 °C, involved the decomposition of vinyl alcohol. The second stage of degradation occurred between temperatures of approximately 421 °C and 485 °C and involved the fragmentation of the ethylene component [32,33]. After the grafting of BTCA, the weight of the first degradation stage decreased, which was due to the consumption of vinyl alcohol by the esterification. The zeta potential of NFAs reduced from −0.24 to −9.50 mV (Figure S3), indicating that the modification would enhance the electronegativity of NFAs. As shown in Figure 1d, the EVOH/BTCA NFAs could freely stand on the top of green bristlegrass, demonstrating the lightweight properties of EVOH/BTCA NFAs.

### 3.2. Mechanical and Morphologies Properties of EVOH/BTCA NFAs

**Figure 2:**
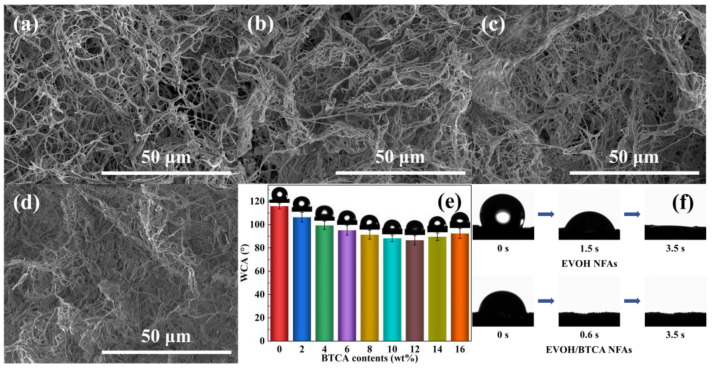
SEM images of (**a**) EVOH NFAs; EVOH/BTCA NFAs with BTCA contents of (**b**) 4, (**c**) 10, and (**d**) 16 wt%; (**e**) the initial WCAs of EVOH/BTCA NFAs; and (**f**) photographs displaying the dynamic permeation process of water on EVOH and EVOH/BTCA NFAs.

As presented in Figure 2a, the EVOH NFAs exhibited a highly interconnected network of fibrous structure. After introducing BTCA contents of 4 wt% into the modification solutions, EVOH/BTCA NFAs presented a slight compact bonding structure among EVOH nanofibers (Figure 2b), which was due to the esterification under PPA, which served as a catalyst. When the BTCA contents increased to 10 wt%, although the pore size decreased, NFAs still maintained the characteristics of a connected porous network structure (Figure 2c). However, after we further increased the BTCA contents to 16 wt%, a cross-linked dense fibrous layer was generated in NFAs (Figure 2d). The total volume of EVOH/BTCA NFAs decreased as the BTCA content increased. EVOH/BTCA NFAs with a BTCA concentration of 16 wt% displayed deformation and lost their regular shape (Figure S4). Therefore, the BTCA loading content would affect the morphologies of NFAs.

The absorption characteristics of aerogels are considerably affected by the surface wettability. The water contact angle (WCA) was used to assess the surface wettability of EVOH/BTCA NFAs. Figure 2e shows that the initial WCA of the EVOH NFAs is 115.9°. After modification with BTCA, the initial WCA decreased to 106.4°, indicating the improvement in hydrophilicity. This is due to the augmentation of hydrophilic groups (-OOH) on the surface by introducing BTCA [34]. With increasing BTCA contents, the WCA decreased gradually until it reached 86.6° at the BTCA contents of 12 wt%. However, the WCA increased instead with a further increase in BTCA contents, which was ascribed to the fact that the dense fibrous layers that formed at high BTCA contents prevent water droplets from penetrating NFAs. As displayed in Figure 2f, the water droplet was absorbed in 3.5 s, confirming that aerogels possess good water wetting surfaces. After grafting BTCA, the water droplet was absorbed by EVOH/BTCA NFAs in less than 1 s. The results are consistent with those of the previous literature [27,35].

**Figure 3:**
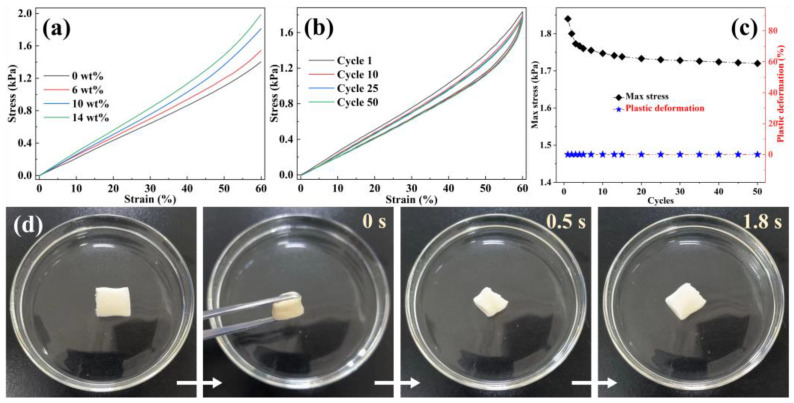
(**a**) Underwater compressive stress–strain curves (ε = 60%), (**b**) underwater cyclic stress–strain curves of EVOH/BTCA (BTCA content is 10 wt%) NFAs at a strain of 60%, (**c**) compressive strength and plastic deformation of EVOH/BTCA NFAs during the cyclic compression process, and (**d**) photographs of the underwater shape-memory properties of EVOH/BTCA NFAs.

Protein absorption and purification processes are conducted in aqueous environments; thus, underwater compression tests were used to assess their practical application performances. Figure 3a displays the stress–strain graphs of EVOH/BTCA NFAs. The observed deformation may be divided into two separate regions: a linear elastic deformation zone for stresses below 20% and a densification region for strains beyond 20% [36]. The underwater compressive strength increased, with increasing BTCA contents, indicating that grafting is beneficial for improving the stiffness of NFAs. The absorption and separation media are usually subjected to cyclic compression and decompression during actual applications. The underwater compressive fatigue resistance of EVOH/BTCA NFAs was further measured through a cyclic compression test. As displayed in Figure 3b, EVOH/BTCA NFAs (BTCA content is 10 wt%) were compressed repeatedly. The hysteresis loops have been identified throughout 50 compressive cycles, resulting from the energy dissipation of EVOH/BTCA NFAs under cyclic compression. A slight reduction in compressive strength and almost 0% plastic deformation were shown after multicycle compression (Figure 3c), demonstrating the good underwater compressive fatigue resistance of EVOH/BTCA NFAs. Attributed to the good underwater compressive properties, EVOH/BTCA NFAs also exhibited shape-memory features. As presented in Figure 3d, squeezed EVOH/BTCA NFAs were thrown into water and then recovered rapidly to their initial shape within 1.8 s. However, it takes six seconds for squeezed EVOH NFAs to return to their original shape (Figure S5). The results clarified that grafting can enhance the underwater shape-memory properties of NFAs. Their superior underwater compressive properties guarantee the long-term utility of EVOH/BTCA NFAs for protein absorption and separation.

### 3.3. Optimizing Protein Absorption on EVOH/BTCA NFAs

**Figure 4:**
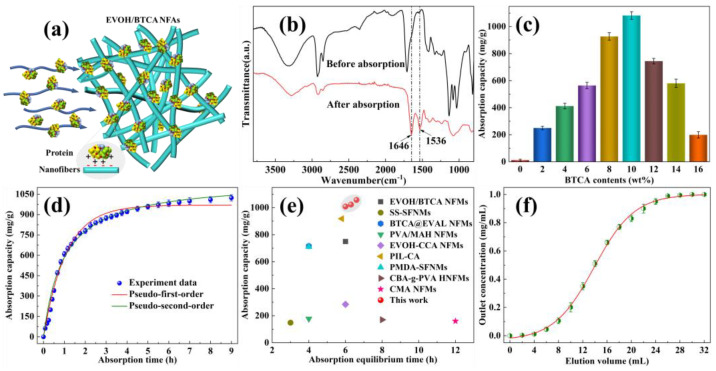
(**a**) A representation to show the process of positively charged proteins being absorbed. (**b**) ATR-FTIR spectra show changes in EVOH/BTCA NFAs before and after lysozyme absorption, (**c**) the capacity of EVOH/BTCA NFAs to absorb substances, (**d**) the capacity of EVOH/BTCA NFAs to absorb substances at different contact times, (**e**) a comparison of the absorbent’s ability to adsorb proteins and the time it takes to reach equilibrium absorption, and (**f**) graphs showing the breakthrough curves of protein absorption for EVOH/BTCA NFAs.

As displayed in Figure 4a, the highly interconnected porous network structures of EVOH/BTCA NFAs can facilitate the rapid penetration of proteins into EVOH/BTCA NFAs and contact with the absorption ligands. The positively charged proteins in the solution are immobilized on the surfaces of aerogels through electrostatic interaction, which can realize protein absorption and separation. The component of protein that was absorbed was lysozyme. The absorption of lysozyme resulted in two distinct peaks corresponding to amide groups, particularly at 1536 and 1646 cm^−1^, as evidenced in the absorption spectra (Figure 4b). The ATR-FTIR results proved that EVOH/BTCA NFAs possessed lysozyme capture performance. The absorption capability of EVOH/BTCA NFAs is illustrated in Figure 4c. There was a linear relationship between the concentration of BTCA and the absorption capacity. The EVOH/BTCA NFAs exhibited a maximum absorption capacity of 1082.13 mg/g when the BTCA concentration was 10 wt%. This value was roughly 20 times greater than that of commercially available membranes [37]. The remarkable protein absorption capability of EVOH/BTCA NFAs can be due to their highly linked network and plentiful absorption ligands. The absorption effectiveness of EVOH/BTCA NFAs decreased as the BTCA concentration increased, mainly because the thick fibrous networks reduced the effective contact area [27].

The influence of absorption time on the absorption capacity was investigated to evaluate their kinetic absorption performance. As displayed in Figure 4d, the absorption capacity increased rapidly first and then reached an equilibrium value of 1010.53 mg/g within 6 h. The absorption kinetics have been investigated using the pseudo-first-order and pseudo-second-order theories. According to the results shown in Table 1, the correlation coefficients (R^2^) for the pseudo-first-order and pseudo-second-order models were 0.98804 and 0.99108, respectively. Therefore, the procedure for the absorption of lysozyme was accurately described by the pseudo-second-order model. Furthermore, the calculated theoretical pseudo-second-order absorption capacity could be as high as 1255.86 mg/g, demonstrating that the absorption capacity could continuously increase by optimizing the structural properties of EVOH/BTCA NFAs. In comparison to most reported carboxylated nanofiber-based protein absorbents, EVOH/BTCA NFAs possessed a superior integrated protein absorption performance [6,38,39] (Figure 4e). The dynamic absorption properties are essential evaluation factors for practical applications. EVOH/BTCA NFAs (10 mm thickness) were assembled into chromatography columns, and then the lysozyme solution penetrated the aerogels via gravity. The typical absorption breakthrough curves are shown in Figure 4d, and the outlet concentration increased to the original concentration of lysozyme solution with the increase in the elution volume. The determined dynamic absorption capacity achieved a maximum of 716.85 mg/g, representing about 66% of the maximal static equilibrium absorption capacity. The excellent dynamic protein absorption properties of EVOH/BTCA NFAs were mainly attributed to their stable and highly carboxylated three-dimensional porous structures [19,40].

**Figure 5:**
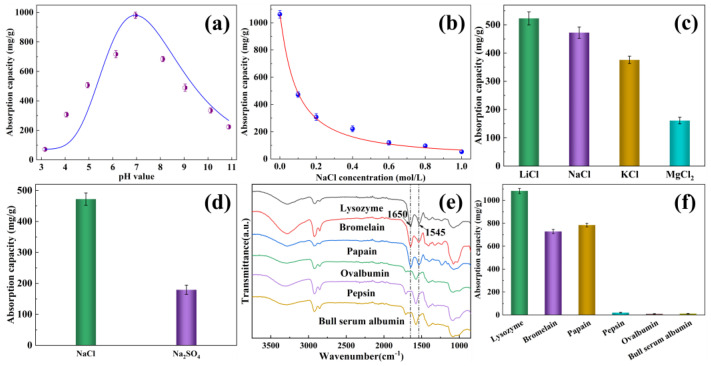
This figure illustrates the impact of several factors on the absorption capacity for EVOH/BTCA NFAs, like (**a**) pH value, (**b**) ionic strength, (**c**) cationic species, and (**d**) anionic species. Additionally, in (**e**), we can see the ATR-FTIR spectra of EVOH/BTCA NFAs after absorbing various proteins, and (**f**) shows the absorption capacities of EVOH/BTCA NFAs for different proteins.

Besides the physicochemical properties of EVOH/BTCA NFAs, the parameters of protein solution have an influence on the protein absorption properties in actual applications. The optimal parameters of protein solution were explored for actual applications of EVOH/BTCA NFAs. As illustrated in Figure 5a, the absorption capacity was relatively low at a pH of about 3, then increased largely to a maximum of about 980.41 mg/g with the increase in the pH value to about 7.0. This is due to the fact that more hydronium ions would be ionized from carboxyl groups on EVOH/BTCA NFAs to form more adsorption ligands [41]. However, the absorption capacity decreased when continuously increasing the pH value, which might be attributed to the reduction in positive charges on lysozyme at a high pH value [28]. Therefore, when the charges of EVOH/BTCA NFAs and lysozyme reached an optimal synergistic effect, the maximum absorption capacity would be achieved. The pH value of about 7.0 was used for the following experiments. The lysozyme solutions with various NaCl contents were taken to study the effects of ionic strength on absorption properties. The absorption capacity in different ionic strengths was shown in Figure 5b. With increasing NaCl concentrations, the absorption capacity decreased largely. When the NaCl concentration increased to 1.0 mol/L, EVOH/BTCA NFAs could not absorb lysozyme. The decrease in electrostatic contact force between aerogels and lysozyme may be attributed to the presence of more salt ions [42].

The ionic species also have significant effects on the absorption performance. EVOH/BTCA NFAs were soaked in the lysozyme solution with the same concentration and different ionic species. As presented in Figure 5c, the absorption capacity in the presence of KCl was much lower than that of in the presence of LiCl and NaCl, suggesting that ions with larger radii would have more substantial shielding effects on absorption. The absorption capacity in the presence of MgCl_2_ was the lowest, indicating that ions with higher charges have greater effects on the absorption performance. Besides cationic species, the absorption performance of EVOH/BTCA NFAs was also inhibited by the anionic species (Figure 5d).

Proteins with different isoelectric points and surface charges were selected to study selective absorption. As presented in Figure 5e, the amide groups’ peaks (at 1545 and 1650 cm^−1^, respectively) were observed on NFAs after the absorption of positively charged proteins (lysozyme, papain, and bromelain) [43]. Nevertheless, the NFAs did not exhibit any peaks corresponding to amide groups following the absorption of negatively charged proteins such as ovalbumin, pepsin, and bull serum albumin. The results indicated that EVOH/BTCA NFAs can be used to extract and separate positively charged proteins from complex biological substrates. EVOH/BTCA NFAs possessed good absorption performances with positively charged proteins, including lysozyme, bromelain, and papain, with capacities of 1082.13, 728.59, and 784.7 mg/g, respectively (Figure 5f). The variation in absorption capacity can be mainly attributed to differences in protein molecule size and surface charges. The smaller molecule size and higher surface charges would lead to a greater absorption capacity [35,44]. The negatively charged proteins would not be absorbed by EVOH/BTCA NFAs.

**Figure 6:**
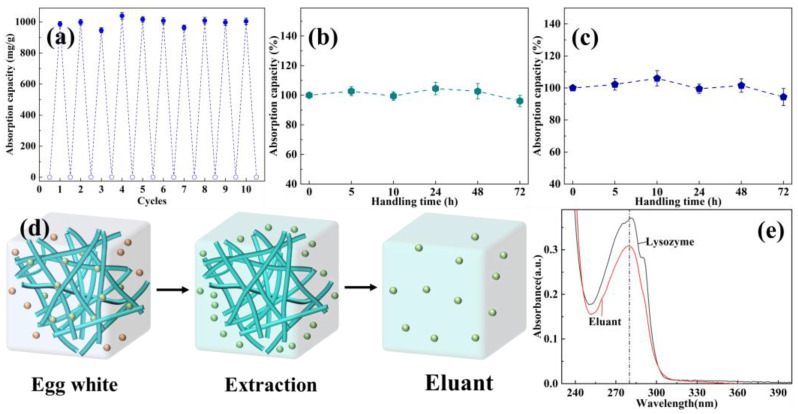
(**a**) The recycling protein adsorption capabilities of EVOH/BTCA NFAs, the absorption performances after treatment with (**b**) acid and (**c**) alkaline buffer, (**d**) the schematic of lysozyme extraction process, and (**e**) the UV absorbance curves of lysozyme and eluent.

The reusability of absorbents is an extremely important index for actual applications. As displayed in Figure 6a, EVOH/BTCA NFAs could show a relatively stable net absorption capacity of about the same as the initial net absorption capacity after 10 cycles, demonstrating the good reusability of EVOH/BTCA NFAs. Generally speaking, absorbents would bear acid and alkaline conditions during protein absorption and elution processes; therefore, protein absorbents should be required to possess acid and alkaline resistance [45]. EVOH/BTCA NFAs were immersed into alkaline and acid buffer solutions to test their acid and alkaline resistance. As presented in Figure 6b,c, the absorption performance almost did not change even after being treated with acid and alkaline buffer solutions for 72 h, indicating that EVOH/BTCA NFAs possess good acid and alkaline resistance. The results could be ascribed to the stable physicochemical structures of NFAs and robust ester bonds between EVOH and BTCA.

Furthermore, the obtained EVOH/BTCA NFAs were taken to extract lysozyme from egg white to evaluate their actual application performance. The egg white solutions were prepared through mixing egg white with PBS, which then were centrifuged for 30 min to form pellucid egg white solutions. The extraction process of lysozyme is shown in Figure 6d. The eluant was collected through the desorption of absorbed EVOH/BTCA NFAs with NaCl solutions and characterized using a UV-vis spectrophotometer. The UV absorbance curve of eluent is basically consistent with that of lysozyme (Figure 6e), proving the extraction and separation of EVOH/BTCA NFAs.

## 4. Conclusions

In conclusion, a facile and universal approach is developed to design and construct highly interconnected three-dimensional protein absorbents. BTCA-functionalized EVOH nanofibrous aerogels (EVOH/BTCA NFAs) were obtained through combining freeze-drying and impregnation processes. The functionalization of BTCA was demonstrated by ATR-FTIR, TGA, and zeta potential results. The BTCA contents significantly affect the physicochemical properties of EVOH/BTCA NFAs, including microstructure, surface wettability, mechanical properties, and absorption performance. The obtained EVOH/BTCA NFAs displayed good underwater elasticity, compressive fatigue resistance, and shape-memory properties. The EVOH/BTCA NFAs functionalized with 10 wt% possessed an outstanding static protein adsorption capability of 1082.13 mg/g within 6 h and a high dynamic adsorption capacity of 716.85 mg/g, which were better than those of most nanofibrous-based protein absorbents. Furthermore, EVOH/BTCA NFAs presented unique selectivity performance, good reversibility, and acid and alkali resistance. The EVOH/BTCA NFAs could extract lysozyme from egg white solution, indicating their potential actual application. Taking in account the low cost of the preparation process and the high performance of protein absorption, we expect that the protein absorbents presented in this study provide a new choice for the design of media in the fields of biological purification.

## Figures and Tables

**Table 1:** Pseudo-first- and pseudo-second-order kinetic parameters.

Kinetic Models	qe (mg/g)	K (min^−1^)	R^2^
Pseudo-first-order model	936.61	0.02	0.98804
Pseudo-second-order model	1255.86	2.20	0.99108

## Data Availability

The original contributions presented in the study are included in the article/Supplementary Material, further inquiries can be directed to the corresponding author.

## Supplementary Materials

The following supporting information can be downloaded via this link: https://www.mdpi.com/article/10.3390/polym16091270/s1, Figure S1: FE-SEM images of EVOH NFAs; Figure S2: Schematic of carboxyl grafting process; Figure S3: The zeta potential of EVOH NFAs and EVOH/BTCA NFAs; Figure S4: Photographs of EVOH/BTCA NFAs with various BTCA contents (the BTCA content from left to right is 0, 4, 10 and 16 wt%); Figure S5: Photographs of the underwater shape-memory properties of EVOH/BTCA NFAs.
