# Supplementary material for: Butane Tetracarboxylic Acid Grafted on Polymeric Nanofibrous Aerogels for Highly Efficient Protein Absorption and Separation
Source: Polymers (Basel). 2024 May 2;16(9):1270. doi: 10.3390/polym16091270 (PMC11085302; doi:10.3390/polym16091270)
Supplement: Supplementary file 1 [file polymers-16-01270-s001.zip › polymers-2927527-supplementary.pdf]

Supplementary Material of

Butane tetracarboxylic acid grafted on polymeric nanofibrous aerogels for highly efficient protein absorption and separation

Jianwei Lu*, Yangang Jiang, Yufei Qiao, Zihao Wen, Zhengjin Luo, Mukhtar Ahmed, Amjad Ali, Li Guo*

School of Materials Science and Engineering, Jiangsu University, Zhenjiang, 212013, China

Department of Zoology, College of Science, King Saud University, P. O. Box 2455, Riyadh 11451, Kingdom of Saudi Arabia

* Correspondence: jianwei@ujs.edu.cn (J.L.); liguo@ujs.edu.cn (L.G.)

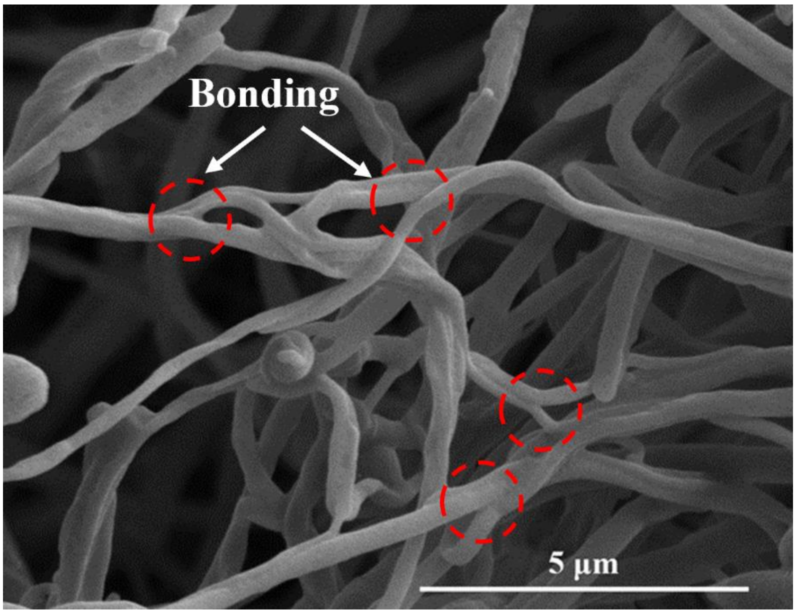

Figure S1. FE-SEM images of EVOH NFAs

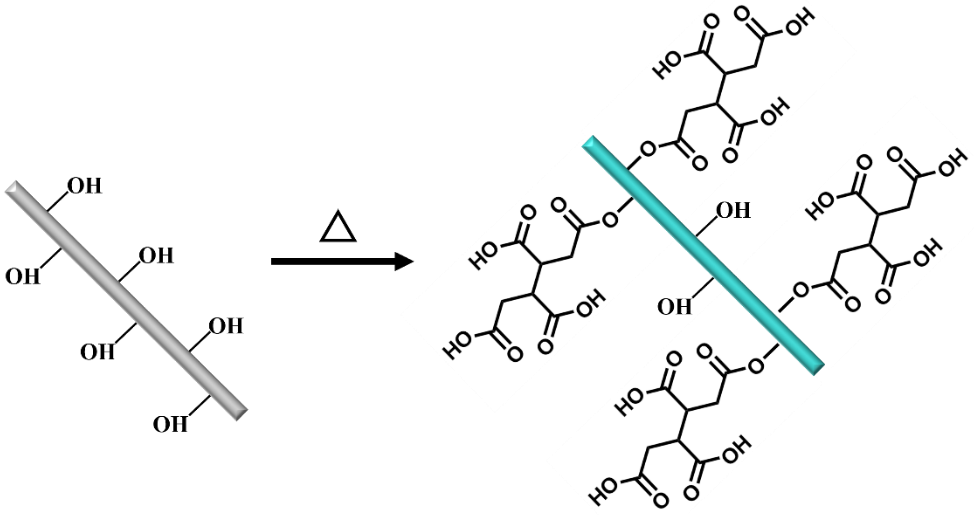

Figure S2. Schematic of carboxyl grafting process

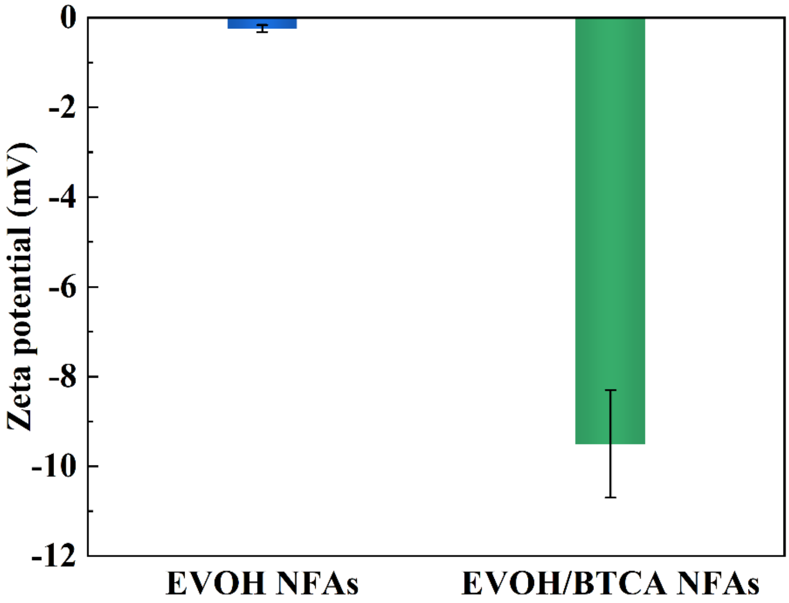

Figure S3. The zeta potential of EVOH NFAs and EVOH/BTCA NFAs

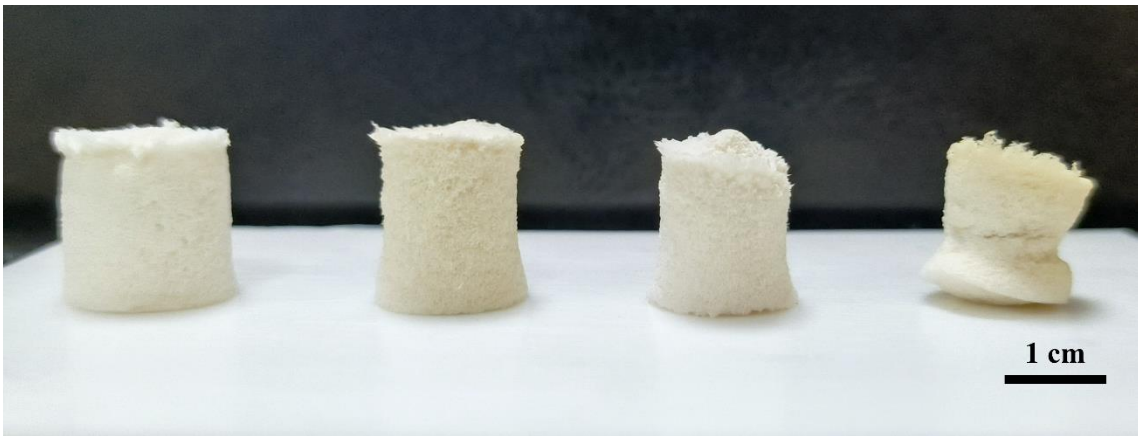

Figure S4. Photographs of EVOH/BTCA NFAs with various BTCA contents (the BTCA content from left to right is 0, 4, 10 and 16 wt%)

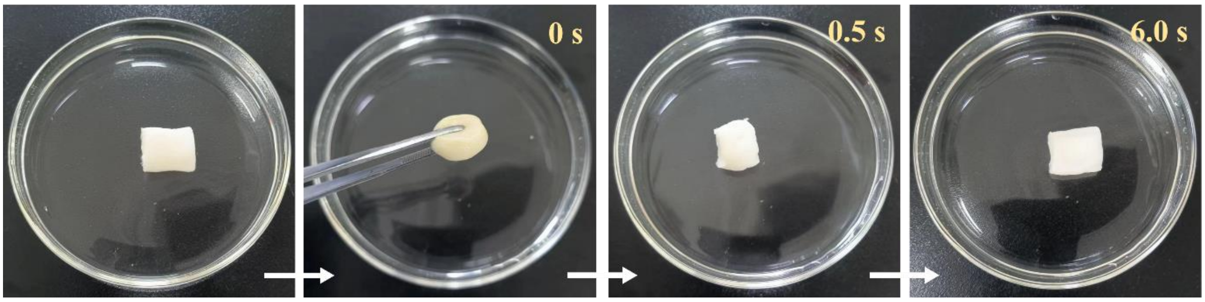

Figure S5. Photographs of the underwater shape-memory properties of EVOH/BTCA NFAs
